# Advances in Mobile Health for Inflammatory Bowel Disease

**DOI:** 10.3390/diagnostics13010037

**Published:** 2022-12-23

**Authors:** Ellen J. Spartz, Lauren DeDecker, Danny Le, Laleh Jalilian, Berkeley N. Limketkai

**Affiliations:** 1 Center for Inflammatory Bowel Diseases, Division of Digestive Diseases, David Geffen School of Medicine at UCLA, Los Angeles, CA 90095, USA; 2 David Geffen School of Medicine at UCLA, Los Angeles, CA 90095, USA; 3 Department of Anesthesiology, David Geffen School of Medicine, Los Angeles, CA 90095, USA

**Keywords:** mobile health, telemedicine, inflammatory bowel disease, Crohn’s disease, ulcerative colitis

## Abstract

Mobile health has the potential to transform the management of chronic illnesses, expanding treatment from a purely clinic-based approach to a more patient-centered delivery of care. For patients with inflammatory bowel disease (IBD), a condition characterized by a relapsing and remitting course, adoption of mobile health strategies can promote improved quality of care delivery and clinical outcomes. Benefits of mobile health applications for IBD include tracking symptoms to guide disease management, coordinating data exchange across clinical care providers, increasing communication between patients and the care team, and providing educational materials to increase patient engagement and satisfaction. In this review, we present the current offerings for telemedicine systems and mobile applications designed for patients with IBD and discuss the potential advantages and limitations of utilizing mobile health in the care of these patients.

## 1. Introduction

The dawn of mobile health arose from several fundamental shifts in the role of technology in society. For one, the advent and eventual ubiquity of smartphones heralded an era of constant connectivity. Computing technology and the network infrastructure advanced enough to allow increased portability of highly capable devices and deployment at scale to the masses. The synergistic impact of these changes would essentially disrupt every facet of life, from education and social interactions to shopping and navigation. The aftershocks have transformed healthcare delivery, a domain that traditionally has lagged in adoption of emerging technologies compared to other industries.

Fundamentally, telemedicine encompasses the use of technology for the transmission of data relevant to the provision of healthcare remotely [1]. Teleconferencing systems enable live, remote interactions between patient and provider outside the traditional framework of an in-person consultation, while messaging tools allow asynchronous electronic communication. Mobile health, on the other hand, augments telemedicine through use of portable devices that can be used for data transmission and for remote data processing [2]. For example, wireless sensors and smartphone applications provide a platform for remote monitoring of pre-defined health parameters. More integrated platforms directly feed health data into the electronic health record (EHR) that can be coupled with expert systems and decision-support tools to semi-automate review and interpretation of the vast array of transmitted data. These suites of applications thus allow continual monitoring and management of patients’ health beyond discrete ambulatory clinic visits or hospitalizations. These technologies additionally facilitate self-care, in which the consumer interacts directly with mobile apps without any intervention by their healthcare provider. Purported benefits of telemedicine and mobile health range from increasing patient access to specialized healthcare, reducing costs, and improving quality of care [1]. Currently, there are many smartphone and web-based applications that have been developed for mobile health, some of which have been clinically studied, yet remain sparse and sporadic in implementation. Further insight and investigation are needed to better understand the impact of mobile health on clinical outcomes and to identify factors that affect mobile health utilization in our healthcare systems. This review presents the features, opportunities, and challenges with mobile health in the context of inflammatory bowel disease (IBD).

## 2. Telemedicine Visits

Prior to 2020, use of telemedicine visits (televisits) in the United States was very limited, largely due to lack of deployed technical infrastructure (including adequate security measures), clinical operational workflows, and insurance coverage. However, the coronavirus disease 2019 (COVID-19) pandemic forced the very rapid adoption of televisits to reduce disruptions in clinical care delivery. Now, after the broad deployment of technical infrastructure and experience, televisits have become increasingly popular in the routine care of patients with inflammatory bowel disease (IBD), even replacing traditional face-to-face visits for many patients. In the United States, audiovisual and telephone visits are currently the most common visit modalities for remote management of patients with IBD [3]. Common audiovisual platforms include those fully integrated into the EHR (e.g., EPIC) or non-integrated platforms, including Zoom, Skype, FaceTime, Google Meet, Microsoft Teams, WeChat, and WhatsApp [3].

Although televisits are unable to replace diagnostic appointments requiring physical, endoscopic, and radiographic examinations, televisits have a wide range of benefits for patients with IBD. Routine follow-up care to monitor or collect diagnostic information can be done through televisits, which can lead to greater convenience for patients and more willingness to engage in frequent check-ups. This feature is particularly useful for conditions, such as IBD, where the clinical course can rapidly change and requires close monitoring. Compared with traditional in-person visits, televisits can be less disruptive for patients with IBD, who might already experience challenges with loss of work and school productivity [4]. Additionally, finding gastroenterologists who are experienced in IBD management is time and cost intensive [5]; televisits have increased access to subspecialists for those who live in more remote areas or otherwise are limited by geographical distance. A study at Dartmouth-Hitchcock Medical Center found that 81% of patients with IBD who participated in telehealth visits lived greater than 25 miles away from their gastroenterologists. Compared to standard in-person visits, patients who met via telemedicine reported time savings of half a day to a full day of time from not having to commute to and from the medical office. Financially, these televisits also saved patients on average $62 per visit [6]. As such, televisits may also help to alleviate the geographical and socioeconomic disparities in access to IBD care.

Televisits may save patients time and money, yet patient satisfaction and quality of healthcare visits must also be evaluated. A study at the University of Maryland demonstrated that 90% of patients with IBD believed all their concerns were addressed [7]. Similarly, at Dartmouth-Hitchcock Medical Center, 91% of patients endorsed understanding their IBD state after the televisit and 78% demonstrated clear understanding of their doctor’s recommendations. The study also found that there were no differences in usage of steroids, biologic therapy, or narcotics [6]. In a systematic review of 17 randomized controlled trials (RCTs) with 2571 patients who received either telemedicine visits or the standard of care, patients in the telemedicine group had higher quality of life and lower number of clinic visits [8]. Additionally, no differences were found in disease activity, remission rates, patient satisfaction, or depression. These findings suggest non-inferiority of IBD televisits compared to in-person visits. In fact, 70% of patients highly rated video consults and preferred this modality over traditional visits [8]. 

Despite many advantages, there are limitations to televisits for patients with IBD. Disparities in access to reliable broadband connectivity or technical literacy can exacerbate health disparities. In 2019, 25% of the United States population lacked home broadband, demonstrating a significant digital divide [9]. Bridging this gap will be important in the care of our patients with IBD. Some interventions have included tablet loan programs, WiFi hot spot identifiers, and discounts on Internet plans for lower-income families [10,11]. Reimbursement inequities for televisits is also a concern, as only half of the states require equal reimbursement of televisits compared to in-person consultations [7]. Billing considerations were already a major barrier to broad adoption of televisits prior to the COVID-19 pandemic.

## 3. Remote Monitoring 

Remote monitoring provides additional opportunities to manage patients with IBD beyond clinic visits. In a traditional clinic model, clinicians only assess symptoms in discrete intervals that are subject to the scheduling constraints of both patients and providers. This provides few opportunities for clinicians to assess patients’ response to therapy. With long and varying intervals between visits, patients may recall their symptoms and symptom severity with poor accuracy. Indeed, with specific regard to gastrointestinal symptoms, questionnaires that rely on recall of symptoms show poor concordance with symptoms collected in diaries [12]. Remote monitoring is one such solution to increase the amount and perhaps the accuracy of symptom surveillance, which can be useful to assess response to therapy as well as identify adverse effects. Several RCTs have studied the impact of remote monitoring on outcomes, such as disease activity, healthcare utilization, and quality of life (Table 1). 

The Telemonitoring of Crohn’s disease and Ulcerative colitis (TECCU) trial involved 3 arms randomized to receiving nursing-assisted telephone care, using a mobile application, or neither (control group). Patients in the mobile application arm reported symptoms through the application; in turn, they received educational materials, reminders, and treatment guidelines. Results demonstrated that disease activity and remission status improved the most with the app-monitored group. There were also fewer phone calls and outpatient visits in the app-monitored group [13].

Another study was the home telemanagement system for ulcerative colitis (UC HAT). Patients were randomized to UC HAT or best available care (BAC) for 12 months. Patients involved in the UC HAT platform answered questions about disease activity, adherence to therapy, side effects, and measured their weight weekly. An educational curriculum was also provided. In intention-to-treat analyses, the study found that compared to BAC, UC HAT did not improve disease activity, quality of life, or adherence at 1-year follow-up. There was a higher rate of attrition of patients in the UC HAT arm compared to BAC arm. However, when data were adjusted for baseline disease-specific quality of life scores, completers of UC HAT had decreased disease activity scores at 12 months and a significant increase in quality of life compared to BAC-completers [14].

The HealthPromise study randomized patients to use a remote monitoring application or an education-only control application. Information collected was integrated into the EHR [15]. Patients updated quality report cards in the app biweekly, tracking their symptoms and measure of quality of life. Providers received reminder/alerts about patient status. Patients who used the HealthPromise platform had improved quality of life measures at 6-month follow-up [16]. 

MyIBDcoach was a RCT that involved weekly or monthly monitoring modules that tracked disease activity, treatment compliance, medication side effects, smoking, quality of life, and anxiety and depression symptoms. When activity thresholds were met, an auto-alert was sent to healthcare providers. MyIBDcoach also integrated a 3-question nutritional assessment every 3 months to screen for malnutrition. The mean number of flares, corticosteroid courses, emergency visits, and surgeries did not differ between groups. However, there was significantly improved treatment adherence, fewer outpatient visits and phone calls, and 50% fewer hospitalizations, which overall lowered IBD-related care costs. Patients were very satisfied, with 94% using the app at 1 year [17].

eIBD is another telemonitoring system that was originally used for IBD patients at UCLA. The app offered appointment reminders, medication trackers, and a healthcare provider portal where patients could message their care team. Patients answered surveys regarding their disease activity and quality of life, with certain responses triggering clinical provider attention. The questionnaires were found to correlate with the Harvey-Bradshaw Index (HBI) for Crohn’s disease (CD) and partial Mayo scores for ulcerative colitis (UC). Other features of the app included modules to track exercise and nutrition. A pilot study of the app found that users had fewer endoscopies, decreased healthcare utilization, and decreased long-term steroid use. Around half of users found the app easy to use and felt an improvement in their disease control [18].

Together, these studies demonstrate various implementations of remote monitoring systems that can be used to track disease activity and patient outcomes. Such systems have the potential to be integrated into EHRs and to involve an automated alert process. Further studies are warranted to validate findings such as decreased disease burden, increased treatment adherence, decreased hospitalizations and outpatient visits, decreased healthcare costs, improved quality of life, and patient satisfaction. 

## 4. Remote Management 

Treatment for IBD patients includes both induction and maintenance therapy. Remote management offers one possible solution to engage patients during both phases but particularly during maintenance therapy. Beyond monitoring symptoms, remote management expands the opportunity for clinicians to enact care plans for their patients beyond traditional clinic visit intervals. This can be achieved through several different platforms including text messages, emails, websites, and mobile applications. Similar to remote monitoring, remote management allows for individualized care, promotes patient engagement in their own care, and can potentially reduce outpatient clinic visits and hospital admissions. Remote management takes remote monitoring one step further: the collection of symptoms, reported side effects, and laboratory data can be used to determine the next steps of management. Management decisions can include type of therapy as well as the strength (dosage) and duration. Remote management can also prompt patients when they should seek in-person care in the clinic or present to a hospital. A few studies have examined the use of remote management with IBD patients and its impact on outcomes such as disease activity, hospitalizations, and quality of life (Table 1). Here, we discuss two prominent examples of remote management platforms that have been studied for patients with IBD.

The TELE-IBD trial randomized patients to remote management of symptoms (monitoring via text messages) or standard care. The design involved a mobile phone for participants and a decision support server and website for staff and providers. Participants responded to a series of text messages regarding their IBD symptoms. Patients with CD completed a modified HBI. For patients with UC or indeterminate colitis, patients completed the Simple Clinical Colitis Activity Index (SCCAI). Patients also were asked if they experienced any medication side effects. Disease-specific quality of life was assessed with a validated IBD Questionnaire. Based on responses to core questions and activity indices for CD and UC/indeterminate colitis, patients were assigned to disease activity zones: green (remission or mild disease activity), yellow (moderate disease activity), or red (severe disease activity). Providers could then select action plans for different patients depending on their severity zone. The provider could individualize alerts and action plans for each participant. If predetermined criteria were met after testing, action plans were sent to the participant and a nurse received an email alert. The nurse reviewed the information and consulted the provider for management changes. Medication changes were updated within the participant profile. TELE-IBD also designed an educational curriculum, which consisted of messages sent to participants twice per month, including reminders such as when to receive routine vaccinations. Patients enrolled in TELE-IBD experienced a decrease in hospitalizations with an associated increase in non-invasive diagnostic tests, telephone calls, and electronic encounters [19]. No differences were found in disease activity or quality of life between control patients and telemedicine patients. Importantly, attrition rates were the highest in the group that received the most frequent interactions with the TELE-IBD system [19].

The Constant Care platform is a web-based protocol that guided treatment for patients with IBD. Small prospective studies were conducted to test these web-based protocols. Inflammatory burden (IB) was a measure created for this web-based approach, which consisted of a clinical index (HBI for CD or SCCAI for UC) and fecal calprotectin. Patients recorded their disease activity and fecal calprotectin weekly and were thus placed into a traffic light system based on their IB score [20]. One such application of the Constant Care platform included a web-based treatment protocol for dosing mesalamine in patients with UC based on IB score. If the IB score was in the green zone, the platform recommended a treatment dose of 2.4 g/day. If the IB score was in the yellow zone, patients were instructed to increase the dose to 4.8 g/day. If the IB score was in the red zone, patients were instructed to continue the dose at 4.8 g/day but in addition to seek additional medical guidance. The study reported significant improvement in quality of life measures for patients who participated in this web-based treatment [21].

The Constant Care platform also created a web-based treatment protocol for dosing of infliximab, which included patients who were receiving maintenance infliximab. Dosing recommendations were also based on the IB score. If the IB score was in the green zone, no new infusion was scheduled. If patients had an IB score in the yellow or red zone, patients were scheduled to have their next infliximab infusion within 72 h. The study results showed no significant differences in hospitalizations, frequency of surgical interventions, or use of corticosteroids between patients in this web-based treatment protocol versus standard care. However, patients who were treated in the web-based treatment protocol did have a reduced number of infusions with an associated reduction in cost [21]. 

These studies are examples of remote management platforms that can be built upon to deliver healthcare to patients with IBD. Both TELE-IBD and Constant Care platforms collected data through a combination of subjective (i.e., symptom reporting) and objective findings (i.e., laboratory results) and then sorted patients into different categories or “zones” of disease burden. This categorization then either triggered an alert to a provider or to an automated change in management, such as a change in treatment dose. Further implementation of such automated processes, which can be carefully designed based on treatment guidelines, may be beneficial in IBD management. Important considerations include challenges with insurance authorization for more frequent dosing of biologic therapies and the risk of developing anti-drug antibodies with inadequate dosing. 

## 5. Personal Mobile Applications 

Mobile applications provide a unique opportunity to engage patients in their own healthcare. Common mobile application features may include virtual visits, care team messaging, symptom tracking and analysis, surveys/questionnaires, data integration from wearable devices, push notifications for reminders, forums, a community platform, newsfeed for research updates, educational modules, and public restroom locators (Table 2). We provide a non-exhaustive overview of currently available personal mobile applications that have been designed for patients with IBD (Table 3). 

The application OshiHealth is available for in-network patients with Aetna insurance and is designed for patients with IBD, irritable bowel syndrome (IBS), gastroesophageal reflux disorder (GERD), celiac disease, and other chronic gastrointestinal (GI) conditions. The application enables users to have virtual visits with and send messages to the Oshi Health professional team, which includes a gastroenterologist, nurse practitioner, registered dietitian, psychologist, and health coach. Not only can users track their IBD symptoms and wellbeing with weekly surveys, but they can also integrate data from their fitness devices and wearables and receive push notifications to help stay on track with their goals. The app features daily inspiration and education about IBD as well as IBD-friendly recipes and personal stories submitted by users. Users can also ask and review questions that have been answered by GI medical professionals in the Questions and Answers (Q&A) section [22]. 

Most other IBD-related applications do not involve a medical care team. GI Buddy is an application made by the Crohn’s & Colitis Foundation that allows users to track their IBD symptoms, food intake, activity, medications and log their medical appointments for reminders. There is also a “community” function in which users can connect with other patients with IBD [23]. Colitis Diary is a paid symptom tracking app that uses a calendar format in which users can record bowel movements, symptoms, possible triggers, exercise, vitals, medications, medical events and tests/procedures, sleep, weather, diet, and write free text notes. There is also a trending module that shows the users the factors and triggers that may contribute to their symptoms [24]. A free alternative is ColitisTracker, which also uses a calendar format to track symptoms, medications, and free text notes [25]. MyIBDCare helps users to track symptoms, flares, appointments, bowel movements, and their medications. It also features an IBD-related newsfeed and courses for education about sleep, medications, and exercise [26]. 

Other mobile applications reach beyond patients with IBD and encompass more generalized GI-related symptom tracking. CaraCare is a tracking application founded in Germany and was one of the first applications that could be prescribed and reimbursed. The app is aimed at those with IBS and symptoms related to consumption of low fermentable oligo-, di-, monosaccharides, and polyols (FODMAP). The app allows users to track their symptoms, bowel movements, menses, physical activity, sleep, medications, pain, and dietary intake. CaraCare analyzes the user data to reveal what foods were eaten on the user’s best and worst IBS-symptom days. There is a library of information on tips for IBS and low FODMAP recipes. There is also an optional 12-week IBS program to follow low FODMAP diet, with unlimited text chat with a dietitian [27]. A similar alternative is Bowelle, which functions as a food and IBS symptom tracker [28]. 

Several apps also exist solely to record bowel movements. PoopMaster [29] and PoopTracker [30] allow for tracking of bowel movements, amount of blood, and stool form. There are also several apps that help users find public restrooms. WeCantWait, made by the Crohn’s and Colitis Foundation, has a library of over 1.3 million public toilets worldwide and is highly rated by users. It also contains education, community support, and a digital copy of the “I Can’t Wait Restroom Card” to help IBD sufferers access restrooms as quickly and seamlessly as possible. Users can manually enter restrooms and add feedback to existing bathrooms for others [31]. Similar bathroom-finder apps include Flush [32] and Toilet Finder [33]. 

MyCrohnsandColitis application focuses on emotional support for patients. It is one of more highly rated and utilized mobile applications and functions as a social network for those with IBD. Members can create profiles that detail their diagnosis, disease story, and their treatments, and users can post photos and updates. Users can follow other users’ updates and post comments and questions to engage in conversation, as well as add others to their “support team” [34]. Similarly, Bezzy IBD functions as a social media platform and also focuses on emotional support. Users can create profiles and post updates, as well as befriend and send messages to others. There is also an extensive selection of forums for open discussion on topics including “Women with IBD,” “Treatment and Side Effects,” and “Newly Diagnosed”. Users also have access to a library of information on topics including “Living Well,” “Managing IBD,” “Sex and Relationships,” and “Diet and Nutrition” [35].

Some applications focus on educational materials. MyGiHealth has a section of the app includes learning modules on GI symptoms and their causes and management, the anatomy of the GI tract, and United States Food and Drug Administration (FDA)-approved treatments for the most common GI diagnoses. There is also a section on mental health related symptoms and how these symptoms relate to physical GI symptoms and methods for symptom management. Another section includes information on how to think positively using techniques of Cognitive Behavioral Therapy (CBT), accept their symptoms with Acceptance and Commitment Therapy (ACT), practice mindfulness and meditation, and learn about the mind–body connection. Users can also receive curated articles, research updates and news notifications to their app inbox [36]. 

For those who may not have access to a smartphone, ibd.care is a website for both IBD patients and clinical providers. It provides patient-friendly education on the basics of IBD, expert recommendations for IBD treatment, tips for talking to IBD providers, lifestyle recommendations, and how to navigate insurance coverage. Clinical providers can earn Continuing Medical Education (CME) credit by learning about personalizing IBD therapy, FDA-approved treatments, American Gastroenterological Association (AGA) care pathways, shared decision making, and how to execute a prior authorization [37]. 

As described, there exists numerous mobile applications commercially available for patients. Potential benefits include increased understanding of disease burden through accurate symptom tracking, access to educational materials, and emotional support through social networks. Whether or not these applications provide meaningful benefits to patients that translate into better care or increased quality of life remains unstudied. 

## 6. Clinical Outcomes

The benefit of personal mobile applications is unclear, given than most of have been developed commercially and have not been studied in a clinical setting. Of the mobile health platforms that have been clinically studied, there have been mixed results on their impact on clinical outcomes (Table 1). A few studies have demonstrated improvement in quality of life (HealthPromise [16] and Constant Care [21]), while others found no differences (UC HAT [14] and TELE-IBD [19]). One study demonstrated improvement in disease activity (TECCU [13]), while others found no improvement (UC HAT [14], MyIBDCoach [17], TELE-IBD [19]). One study demonstrated decreased use of corticosteroids (eIBD [18]), while others found no difference (MyIBDCoach [17], Constant Care [21]). 

Several studies have investigated the impact of mobile health on healthcare utilization for patients with IBD. Mobile health in IBD management has been shown to result in fewer phone calls and outpatient visits (TECCU [13] and MyIBDCoach [17]), fewer infusions (Constant Care [21]), fewer endoscopies (eIBD [18]), and fewer hospitalizations (MyIBDCoach [17] and TELE-IBD [19]). On the contrary, one study demonstrated an increase in non-invasive diagnostic tests, phone calls, and electronic encounters (TELE-IBD [19]). Further, some studies support no difference in hospitalizations (Constant Care [21]) or emergency department visits (MyIBDCoach [17]). Mobile health use in IBD management has also been shown to reduce healthcare-associated costs (MyIBDCoach [17] and Constant Care [21]). 

The heterogeneity in results suggests a need for further prospective research on the impacts of mobile health in IBD management on healthcare outcomes such as disease activity, quality of life, and healthcare utilization and costs. 

## 7. Potential Challenges

Although advances in technology may allow for adoption of many different aspects of mobile health, there remain many potential challenges and limitations with these methods. Many implementations of mobile health involve emails, text messages, Internet portals, or mobile applications, all of which require Internet access, cellular data, and/or access to personal computers or smartphones. Beyond access, mobile health also requires a degree of technical literacy. These factors create a limitation on the wide accessibility of mobile health, although this may improve over time with advancing technologies and the technical aptitude of younger generations. These factors that lead to differences in access to information technologies is commonly referred to as the digital divide, which can impact people of racial and ethnic minorities, people with mental or physical disabilities, rural populations, people of advanced age, and people of lower socioeconomic status. While there are efforts to bridge the digital divide to increase access of mobile health to underserved populations, it is important to consider a bias towards caring for patients who have accepted digitalization over those who have not, whether by choice or circumstance. As some patients may choose not to participate in mobile health platforms, measures should be taken to ensure equitable care for these patients, for example, maintaining availability of in-person visits and timely communication for those who prefer a more traditional clinic approach. 

With numerous platforms for telemedicine and mobile applications, there is a wide variety for patients and providers to choose from. However, this also detracts from having a centralized and standardized approach for management of IBD patients. With wider adoption of mobile health technologies, it will be notable to observe if certain platforms and/or design features and themes will predominate. As with any creation of a mobile application or technology platform, the design should take into consideration many factors including a user-friendly interface, survey fatigue, and a careful consideration of which data points are most relevant. To encourage frequent and long-standing use of mobile health platforms, platforms must be easy to use. Poor design that is either too confusing to use or has frequent errors will disengage patients. Patient engagement and motivation are important to promote long-term use of mobile health platforms. Thus, patient attrition rates and the reason for attrition are important data points to collect. Patient adherence to mobile health platforms varies across different studies. For example, in TELE-IBD, 74% completed the 1-year study [19]. In the UC HAT study, adherence to the telehealth system was 40% at 6 months [14]. Further studies are warranted to elicit key factors that influence adherence to mobile health technologies. 

The amount of data collected should be carefully considered; data collected too infrequently may lead to insufficient information to impact patient outcomes. On the contrary, too frequent monitoring can become burdensome to patients and lead to disengagement. The sheer volume of data to review and synthesize can also be burdensome to healthcare providers. Ideally, adoption of mobile health platforms should seek to automate processes when possible and integrate support staff when able, such as clerks and nurses, who can help manage and triage alerts and facilitate communication. 

Data privacy is another major area of concern. Data meant for the healthcare provider may be collected and stored by the application manufacturer and could also be shared with third parties. The Health Insurance Portability and Accountability Act (HIPAA) of 1996 is a federal law designed to implement principals that protect privacy, such as the need for data encryption. In addition, the Health Information Technology for Economic and Clinical Health (HITECH) Act of 2009 extends HIPAA to business associates that “create, receive, maintain, or transmit” identifiable health information [38]. Mobile applications or telehealth platforms created by third parties must be HIPAA-compliant, but furthermore, should be proactive about data security to prevent leaks. Patients should feel comfortable sharing their health information on websites or mobile applications or via text or email messages, and these data should be protected. As telehealth expands, it is critical that privacy protections are built into technologies and federally enforced to ensure adequate data privacy for users. 

## 8. Conclusions

In a traditional clinic model, symptom monitoring and changes in management occur at discrete intervals that may range from weeks to many months during outpatient follow-up visits. This model may be suboptimal in the management of certain diseases, such as IBD, that are chronic in nature but often with a proclivity for flares. Advancing technologies has the potential to transform the traditional healthcare model to one that fits our modern age. Televisits can increase accessibility of specialized IBD care to patients in more remote areas. Remote monitoring and management can offer more targeted, individualized, and timely patient care. Further studies are warranted to measure the effectiveness of such platforms on outcomes such as disease remission, quality of life, and healthcare utilization and associated costs. Finally, personal mobile applications provide many additional features that include symptom tracking, educational materials, and emotional support. Together, given the ever-expanding nature of digital technologies and its wider adaptation into our healthcare system, these aspects of mobile health will likely alter the landscape for management of patients with IBD. 

## Figures and Tables

**Table 1:** Clinical Studies on IBD Remote Monitoring and Management.

Platform	Study Design	Outcomes
Telemonitoring of Crohn’s disease and Ulcerative colitis (TECCU) [13]	RCT, *n* = 63 - Report symptoms - Receive education and reminders	- Improvement in disease activity and remission status - Fewer phone calls and outpatient visits
Home telemanagement system for Ulcerative Colitis (UC HAT) [14]	RCT, *n* = 24 - Patients surveyed about disease activity, therapy adherence, side effects - Measure weight weekly - Educational curriculum	- No improvement in disease activity, quality of life, or adherence
HealthPromise [15,16]	RCT, *n* = 320 - Patients update quality report cards biweekly, track symptoms and quality of life - Providers receive reminders and alerts	- Improved quality of life
MyIBDcoach [17]	RCT, *n* = 909 - Patients report disease activity, treatment adherence, side effects, smoking, quality of life, depression and anxiety symptoms - Nutritional assessment	- No difference in mean number of flares, corticosteroid courses, emergency room visits, surgeries - Improved treatment adherence, fewer outpatient visits and phone calls, fewer hospitalizations, lower IBD-related care costs
eIBD [18]	Prospective study UC, *n* = 194 CD, *n* = 217 - Patients surveyed on disease activity, quality of life, exercise, nutrition - Responses trigger alerts to providers	- Fewer endoscopies, decreased healthcare utilization, decreased long-term steroid use
TELE-IBD [19]	RCT, *n* = 348 - Surveyed on symptoms, HBI or SCCAI, medication side effects, quality of life - Patients categorized into green, yellow, or red zones of disease activity - Providers are altered and individualized plans created for each participant - Educational curriculum	- Decrease in hospitalizations - Increase in non-invasive diagnostic tests, phone calls, electronic encounters - No differences in disease activity or quality of life
Constant Care [20,21]	Prospective study Mesalamine, *n* = 94 Infliximab, *n* = 27 - Tracking of disease activity, fecal calprotectin, HBI, or SCCAI - Patients categorized into green, yellow, or red zones of disease activity - Protocolized treatment response for mesalamine or infliximab dosing based on zone of disease activity	Mesalamine dosing: - Significant improvement in quality of life Infliximab dosing: - No difference in hospitalizations, surgical interventions, use of corticosteroids - Reduced number of infusions and associated reduction in cost

**Table 2:** Common Features of IBD Mobile Applications.

Feature	Description
Virtual visit	Users can participate in tele-health visits with a medical clinician, dietician, psychologist and/or health coach
Care team messaging	Users can message their clinical care team
Tracking	Users can log their food intake, abdominal pain, other symptoms, activity level, medications, bowel movements, vitals, tests, procedures, appointments
Survey/questionnaires	Users fill out surveys or pre-determined questionnaires to monitor their disease activity or adherence to treatment plan, which can be viewed by healthcare team
Data integration	Wearable devices, such as activity trackers or smartwatches can integrate data into the app
Push notifications	Users can elect to get phone notifications to help them remember appointments or keep on track of their progress towards goals
Forums/Q + A	Users can ask questions and receive answers from other users or healthcare professionals that can be viewed publicly on the app
Community	Users can interact with others such as via messaging, creating a personal profile, following or friending others, posting comments
Analysis	Analysis of tracking input with suggestions for what various triggers may be for the user’s symptoms
Newsfeed	A display of the latest IBD research relevant to the user’s interest
Education	Modules, research updates, or articles on sleep, IBD medications, lifestyle modification, exercise, IBD as a disease, diet, anatomy, mental health, cognitive behavioral therapy, mindfulness, meditation
Toilet locator	A tool to help the users locate the nearest public restroom

**Table 3:** Personal Mobile Applications for IBD Patients.

Mobile Application	Target Population	Tracking/Features	Education	Cost
OshiHealth [22]	IBD, IBS, GERD, Celiac disease, other	- Telehealth team - Weekly surveys - Fitness devices/ wearables	- General - Nutrition	Available with Aetna insurance
GI Buddy [23]	IBD	- Symptoms - Food intake - Exercise - Medications	N/a	Free
Colitis Diary [24]	IBD	- Symptoms - Bowel movements - Exercise - Medications - Sleep - Diet	N/a	Paid
Colitis Tracker [25]	IBD	- Symptoms - Medications	N/a	Free
MyIBDCare [26]	IBD	- Symptoms - Bowel movements - Medications	- Sleep - Medications - Exercise	Free
CaraCare [27]	IBD, IBS, other	- Symptoms - Bowel movements - Exercise - Sleep - Medications - Diet - Pain	- Diet	Free
Bowelle [28]	IBS, other	- Symptoms - Diet	N/a	Free
PoopMaster [29]	General	- Bowel movements	N/a	Free
PoopTracker [30]	General	- Bowel movements	N/a	Free
WeCantWait [31]	General	- Find public restrooms	N/a	Free
Flush [32]	General	- Find public restrooms	N/a	Free
Toilet Finder [33]	General	- Find public restrooms	N/a	Free
MyCrohnsandColitis [34]	IBD	- Member profiles, forums	- Treatments	Free
BezzyIBD [35]	IBD	- Social media, forums	- Treatments - Lifestyle - Sex and relationships - Nutrition	Free
MyGiHealth [36]	General	N/a	- General - GI tract anatomy - Treatments - Mental health - Research updates	Free
Ibd.care [37]	IBD	N/a	Patients: - IBD basics - IBD treatment - Lifestyle - Insurance Providers: - IBD therapies - Shared decision making - Prior authorizations	Free

## Data Availability

No new data were created or analyzed in this study. Data sharing is not applicable to this article.
